# Apply Machine Learning to Predict Risk for Adolescent Depression in a Cohort of Kenyan Adolescents

**DOI:** 10.3390/healthcare13202620

**Published:** 2025-10-17

**Authors:** Hyungrok Do, Keng-Yen Huang, Sabrina Cheng, Leonard Njeru Njiru, Shilla Mwaniga Mwavua, Anne Atie Obondo, Manasi Kumar

**Affiliations:** 1 Department of Population Health, NYU Grossman School of Medicine, New York, NY 10016, USA; hyungrok.do@nyulangone.org (H.D.); sabrina.cheng@nyulangone.org (S.C.); manasi.kumar@nyulangone.org (M.K.); 2 Ministry of Health, Muranga County, Nairobi P.O. Box 26732-00100, Kenya; leonjeru@gmail.com; 3 Nairobi City County Health Wellness and Nutrition Department, Nairobi P.O. Box 30075-00100, Kenya; smwaniga@yahoo.com; 4 Department of Clinical, Neuro Developmental Psychology, Vrije University, 1081 LA Amsterdam, The Netherlands; 5 Department of Psychiatry, University of Nairobi, Nairobi P.O. Box 19676-00202, Kenya; obondo@uonbi.ac.ke

**Keywords:** machine learning, depression, adolescents, lower-middle-income country, adverse childhood experiences, stress, risk factors

## Abstract

**Background:** Adolescent depression is highly prevalent in low- and middle-income countries (LMICs). Identifying top key risk factors is necessary to inform effective prevention program design. Machine learning (ML) offers a powerful approach to analyze complex multidomain of data to identify the most relevant predictors and estimate risks for mental health problems. This paper applies ML to study risks for adolescent depression to enhance adolescent depression prevention efforts in LMICs. **Methods:** Six ML approaches (e.g., Explainable Boosting Machine, random forests, and XGBoost) were applied to study the risks of depression. Data were drawn from a digital health intervention study conducted in Kenya (year 2024–2025, n = 269). Multiple domains of childhood and adolescent adversity and stress experiences were used to predict adolescent depression (using PHQ9-A). **Findings:** We found that ML was a valuable approach in the early identification of adolescents at risk for depression. Among the six ML approaches applied, the random forest approach outperformed other ML approaches, especially when multiple domains of risks were included. We also found that childhood adversity or home adversity alone were not strong predictors for depression. Adding adolescent stress experiences and community school adversity experiences significantly improves the accuracy and predictability of depression. Using the top-15 and top-20 ranking factors, we achieved 74.8% and 75.1% accuracy in depression prediction, which was similar to the accuracy when all 49 adverse/stress factors were included in the predictive model (78.3%). **Conclusions:** Innovative ML and modern predictive modeling approaches have the potential to transform modern preventive mental health care by better utilizing multidomain data to identify individuals at risk for developing depression early and identify top risk factors (for targeted individuals and/or populations). Findings from ML can inform tailored intervention design to better mitigate risks in order to prevent depression problem development. They can also inform the better utilization of resources to target high-need cases and key determinants, which is particularly useful for LMICs and low-resource settings. This paper illustrates an example of how to move toward this direction. Future research is needed to validate the approach.

## 1. Introduction

Adolescent depression is highly prevalent in low- and middle-income countries (LMICs) [1,2,3,4,5,6] and arises from complex, interacting factors [4,5,6]. Many adolescents in LMICs have a history of childhood maltreatment trauma and experience a range of adversities at home, where a high-poverty context contributes to maltreatment, neglect, and abuse [7,8,9,10]. As they transition from childhood to adolescence, additional stressors emerge, including body image, safe sexual practices, peer and parental conflicts, and challenges emanating from adverse school environments such as large class size, poor oversight, and support from teachers and community-level exposure to harm and violence [11,12,13,14,15]. The carry-over unresolved challenges from childhood cumulatively get added to new emerging adolescent identity problems and other developmental problems contributing to depressive affect and mood regulation problems [16,17]. Although a body of epidemiological research has documented multiple domains of factors (i.e., individual, home, neighborhood/community) contributing to adolescent depression [7,8,9,10], how early adversity, the adolescent developmental period, and stressful school experience interact and lead to the development of depression is not well understood. Identifying key predictors that contribute to adolescent depression can inform effective prevention program design. The goal of this paper is to understand how multiple sources of adversity and stress contribute to, or predict, adolescent depression by using machine learning (ML).

ML is a branch of artificial intelligence (AI) that enables a data-driven approach to learn from data and make predictions or decisions without being explicit about theoretical models [18]. In risk identification, ML has been applied to develop predictive models for mental health problems that not only identify well-known key predictors but also uncover and rank additional important risk factors [19]. For example, it has been applied to predict anxiety, depression, and psychiatric conditions using multi-level predictors [18,19,20]. However, risk prediction research that applies ML to the adolescent population in LMICs is limited. We are interested in applying ML to study how different domains of risks predict adolescent depression, and to identify top-ranking risks that are most relevant to adolescent depression. Our long-term goal is to develop ML-based predictive models that can be applied for practical decisions, enabling early prevention and more effective management of mental health risk and burden in high-need and low-resource LMIC contexts. Given our interest in early prevention, we focus on developing prediction models for the early adolescent period (aged 11–14). The age choice is also based on the evidence that half of mental, neurological, and substance disorder cases develop by age 14 years [3]. This study focuses on a community adolescent sample from one LMIC-Kenya.

This paper aims to apply ML to study two specific research questions.

(1) Examine how four domains of stress and adverse factors predict adolescents’ risk of depression: We used a variety of ML methods and traditional regression approaches to develop predictive models. Four domains of risks were studied separately and jointly. The risk factors included (i) adverse childhood and adolescent experiences (ACEs); (ii) poverty/financial stress; (iii) community and school adverse experiences; and (iv) adolescent developmental stress. *Hypothesis*: (1) The multidomain approach of prediction would have better predictability than a single-domain approach to prediction. (2) The ML approach would have better performance than the traditional parametric linear regression approach, and certain non-linear ML approaches would outperform other ML approaches.
(2) Identify the top-ranking risks that best predict adolescent risks of depression and evaluate their predictive power.

## 2. Materials and Methods

### 2.1. Study Population

The participants came from an adolescent digital health pilot project, the mHealth Toolkit for Screening & Empowering Lives of Youth (mSELY). The mSELY was developed as a universal prevention tool to support adolescent mental health through a single-session interaction session for adolescents Grades 5, 6, and 7 (typically ages 11–14 years). An evaluation study was designed to assess whether the use of mSELY (in comparison to the waitlist control) promotes mental health literacy and prevents the risks of depression in adolescents. For the intervention arm, a tablet was given to adolescents during the session, and adolescents had the opportunity to interact with the Toolkit for self-assessment, self-reflected stress experiences, and learning mental health promotion strategies. Adolescents in the waitlist control did not have the opportunity to reflect on childhood and adolescent stress experiences (the predictors of the study); thus, they were not considered the subject of this study. This study only included adolescents from the intervention arm. Intervention and control adolescents did not differ in sex, grade, mental health literacy, and depression score at baseline, suggesting the representation of the sample.

### 2.2. Data Collection and Study Procedures

The pilot study was conducted in 2024. The intervention arm adolescents were recruited from six schools in Nairobi, Kenya. We selected 2 blocks of schools similar in school size, and each block included 3 schools. Within the block, we randomly assign one school to target Grade 5, one school to target Grade 6, and the other school to target Grade 7. All adolescents from the selected class/grade were eligible and invited to participate in mSELY (a universal prevention program). Each classroom had approximately 50 adolescents. Family and adolescents were invited to group meetings that took place in schools. Teachers and school health teams facilitate family contact. Study information was given to all families and adolescents in the first meeting. Only adolescents and caregivers who agreed and signed the assent and consent participated in the study. After the consent/assent, adolescent participants were first asked to complete the baseline evaluation assessment (a short survey included demographic, health literacy, and PHQ-A questions), followed by the mSELY Toolkit, which includes stress–social–emotional–behavioral self-assessment, psychoeducation, and strategy learning. Both baseline evaluation and toolkit data (collected during the same meeting/session) were included in this study. A total of 278 parents and adolescents from the selected grades consented/assented to participate, and 269 adolescents (97%) provided depression and toolkit data were included in the analytical sample. Nine (3%) consenting/assenting adolescents who did not provide PHQ-A were not included in the ML study. These excluded cases did not differ from those included on sex and age. Follow-up data is collected a year after the first assessment (in 2025), which is ongoing. For this study, only the baseline data were used. We have a plan to validate the predictive model in 2026 when follow-up data are available.

### 2.3. Study Measures

Standardized instruments were used to assess adolescents’ adverse childhood and adolescent experiences. All the measures were based on adolescent self-reports. Assessments were conducted in English because English is the compulsory language of instruction in schools in Kenya.

**Figure 1:**
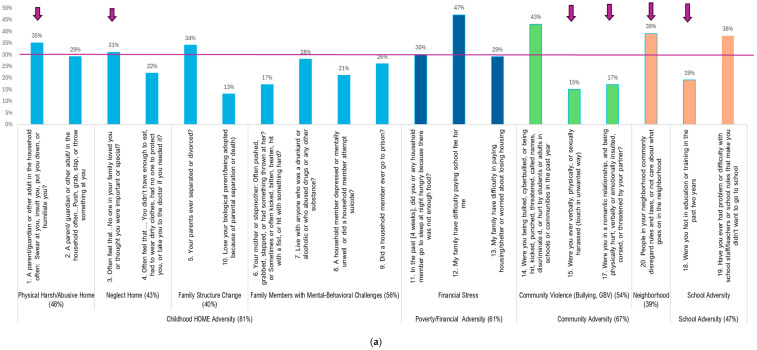

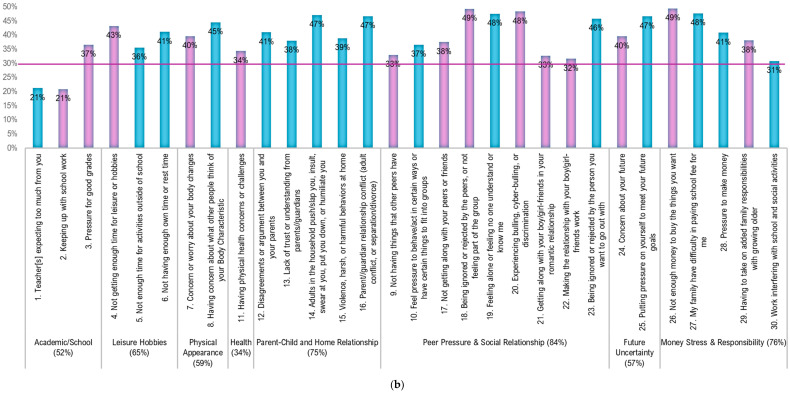
Adversity and stress experiences in Kenyan adolescents. (**a**) Childhood/home, poverty, and community/school-related adversity experiences: % numbers show in the figure are the proportion of adolescents reported “Yes” for the experience. Arrows are those identified as the top 20 ACEs/stress adversity predictors for adolescent depression. (**b**) Adolescent Developmental Stress Experience: % numbers shown in the figure are the proportion of adolescents reported moderately, quite, or very stressful. Purple color bars are those identified as the top 20 ACEs/stress adversity predictors for adolescent depression.

Adverse childhood and adolescent experiences (ACEs) were assessed using an adapted version of the Adverse Childhood Experiences International Questionnaire (ACE-IQ) [10,21]. ACE-IQ assesses an individual’s experiences of childhood adversities at home, such as child maltreatment and other family dysfunction and stressors, and has been validated with a Kenyan sample [21]. To ensure relevance for adolescents in Kenya, we adapted the tool and added 9 items. The adapted version includes the original Home/Family-ACEs (10 areas), and added Poverty-ACEs (3 items), and Community/School ACEs (6 items) (see Figure 1 for item information). Detailed adaptation procedure is described in Appendix A. Adolescents were asked to rate their experiences while growing up (in their earlier and current life) across three domains using a yes/no binary response. Cronbach’s Alpha reliability of the ACEs was 0.78 using the current study sample. Total ACEs score was also positively associated with PHQ-9A (*r* = 51, *p* < 0.001).

Adolescent Development Stress was assessed using an adapted version of the Adolescent Stress Questionnaire (ASQ) [22]. The original ASQ includes 58 items and assesses 10 areas of adolescent stress. Each item (stressor) is rated on a 5-point Likert scale (1 = not at all stressful/or irrelevant to me, 3 = moderately stressful, 5 = very stressful). For this study, we included 30 items relevant to the LMIC context. We also added a physical health stress item, as many adolescents experience physical health problems due to living in poverty. The revised ASQ-K (for Kenya) captures common areas of adolescents’ stress, including academic/schoolwork stress, lack of leisure, physical appearance, peer comparison/comparative, physical health, home relationship, social relationship, romantic relationship, future uncertainty, financial pressure, and emerging adult responsibility stress (see Figure 1b for item information). Detailed adaptation procedure is described in the Appendix A. The Cronbach’s Alpha reliability of the ASQ was 0.90 when using the current study sample. The ASQ mean scale score was positively associated with PHQ-9A (*r* = 0.38, *p* < 0.001).

Adolescent depression was assessed using PHQ-9 for Adolescent (or PHQ-9A) [23], which is a standard PHQ-9 depression screening tool for use in adolescents ages 11–17. PHQ-9A has been validated and used with adolescents in Kenya and LMICs [24,25,26]. Adolescents rate each item on a 0 to 3 scale (0 = not at all, 3 = nearly every day). A total score, the sum of 9 items (range 0–27), was produced. A sum score of 0–4 indicates no or minimal depression (30% of the current Kenya study sample), a score of 5–9 indicates mild depression (36% of the current sample), a score of 10–14 indicates moderate depression (24% for the current sample), a score of 15–19 indicates moderately severe (9% of the current sample), and a score of 20–27 indicates severe depression (<1% of the current sample) [23]. For the current study sample, Cronbach’s Alpha reliability of the PHQ-9A was 0.68. The mean (SD) for the current study sample is 7.71 (4.79).

### 2.4. Statistical Analysis

To study the prediction of four domains of adverse and stress experiences on adolescents’ risk of depression, we used supervised machine learning algorithms. Six ML approaches were carried out, including random forests [27], Bernoulli Naive Bayes Classifier, Support Vector Machines with radial basis function kernels [28], Gradient Boosting Machines [29], Histogram-based Gradient Boosting [30], and Explainable Boosting Machines [31]. We compared six widely used and complementary ML families to balance linear and non-linear modeling capacity, interpretability, and predictive performance. This set represents standard approaches in applied machine learning, ensuring that our findings are not tied to a single modeling assumption and are consistent with common benchmarking practices. Logistic regression was also included as a benchmark given its common use in clinical risk modeling, while ensemble-based methods were evaluated for their potential to capture non-linear associations and interactions among predictors. In the ML model, we first tested models separately for each domain of risks/stress, and we then tested the domains of risk jointly. We tested a total of 7 sets of prediction models (including different domains of predictors): (i) Home-ACE domain only (10 items), (ii) Poverty-ACE domain only (3 items), (iii) Community/School ACEs only (6 items), (iv) Adolescent Developmental Stress (ADS) domain only (30 items); (v) combine two home adversity domains (Home + Poverty) (vi) combine home adversity domains (Home + Poverty) + Community/school domains; (vii) combine all environmental domains (Home + Poverty + Community/school) + ADS domain (49 items). To preserve the item-level information, we conducted the predictive model using the item-level data (instead of using subscales summary score). The depression outcome was modeled using validated binary outcomes of PHQ-9A. We carried out predictive models based on the 10 cut-off, which indicates an individual’s risk of developing moderate to severe depression (score range 10–27). We did not use the more severe clinical cut-off of 15 (moderate to severe depression, score range 15 to 27) because of our interest in early prevention and a small number of clinical samples (<10% of our sample) [32].

All models were implemented in Python 3.10 (https://www.python.org/) using standardized preprocessing pipelines. Hyperparameters were set to default values unless otherwise noted, and random seeds were fixed to ensure reproducibility. We studied the performance of predictive models using repeated K-fold cross-validation with 5 folds and 10 repeats [33]. For each iteration, 80% of the data was used for training and 20% for validation. Within each training split, missing values for continuous predictors were imputed using the training-fold mean, and predictors were standardized by training-fold z-scores. The optimal operating threshold for classification was selected using Youden’s J statistic, calculated on the training split, and the chosen threshold was then applied to the held-out testing fold [34].

Six indicators were used to evaluate model performance: (i) AUC (Area Under the ROC Curve; 0.5 indicates random performance, 1.0 is perfect; values ≥ 0.70 are commonly considered acceptable, ≥0.80 good, and ≥0.90 excellent); (ii) accuracy (overall proportion of correct predictions; in many clinical prediction contexts, ≥70–75% is often viewed as good); (iii) sensitivity (true positive rate); (iv) specificity (true negative rate), noting that a target of ≥70% is typical but thresholds of 60–70% may still be acceptable depending on context; (v) Brier score (mean squared difference between predicted probabilities and observed outcomes; lower values indicate better overall calibration/discrimination balance); and (vi) Expected Calibration Error (ECE), computed as the weighted average absolute difference between predicted probabilities and observed outcome frequencies across probability bins, summarizing calibration quality [35].

For sensitivity and specificity, a threshold of 70% or greater is often targeted, but acceptable thresholds may be set lower (e.g., 60–70%) [35]. In mental health settings, low sensitivity (60–70%) may still be acceptable for providing support but not replace clinical judgment. Higher specificity may also be wanted to reduce false positives, which is important for avoiding unnecessary interventions or worry for the individual [36].

To identify key risk factors (specifically top 20 ranking risks) that best predict adolescent risk of depression, we built on the best ML predictive model (with a higher AUC), and further identified the Top 5, 10, 15, and 20 ranking risks by using Shapley additive explanations (SHAP) [37]. SHAP is a method that breaks down a model’s prediction into contributions from each variable, showing how much each factor pushes the prediction higher or lower. We applied SHAP in two ways. First, we examined global importance, which averages the contributions across all participants to identify the variables that are most influential overall in predicting depression risk. Second, we investigated individual-level explanations, which show the main factors driving the prediction for each person. For each participant, we identified the single most influential predictor (top-1 factor) and calculated the proportion of individuals for whom each variable was dominant. This allowed us to explore whether certain risk factors were consistently the most important across many individuals or whether patterns differed between subgroups. The global analysis provided insight into the strongest risk factors in the overall population, while the individual-level analysis offered personalized profiles that could guide targeted screening and tailored interventions.

In a secondary set of experiments, the ranked list of predictors from SHAP analysis was used to create reduced predictor sets (top 5, 10, 15, and 20 variables). Models were retrained using only these subsets, and predictive performance was reassessed using the same repeated cross-validation strategy. This approach evaluated whether accurate risk stratification could be maintained using a shorter, clinically feasible screening tool based on the most influential variables. All statistical analyses and model training were conducted in Python, utilizing the scikit-learn [30] and SHAP [37] libraries for modeling and interpretability.

## 3. Results

### 3.1. Study Sample Characteristics

Table 1 describes the demographic profile of the study sample and intensity of adversity and stress experiences across four domains. Figure 1a,b characterizes adverse and stress patterns experienced by Kenya adolescents. As shown, this study cohort was 51% male and 49% female; 31% from the 5th grade, 33% from the 6th grade, and 36% from the 7th grade, with a mean age of 12.17 (SD = 1.04) years and 94% Christian. In addition, 81% of adolescents reported experiencing at least one form of home adversity, with a mean count number of ACEs-Home Adversity (out of 10) of 2.55 (SD = 2.08). Poverty/financial adversity was reported by 61% of adolescents, with a mean count number of Poverty/Financial ACEs 1.05 (SD = 1.02) (out of 3). Community-ACEs were experienced by 67% of adolescents, with a mean count of 1.13 (SD = 1.03) (out of 4). School-ACEs were reported by 47% of adolescents, with a mean count number of 0.56 (SD = 0.65) (out of 2). In total, 91% of adolescents reported experiencing at least one area of home, poverty, community, or school adversity, and only 9% reported no adversity experience among the listed adversities in Figure 1a. There were no significant sex differences in these three domains of ACEs.

In developmental stress (Figure 1b), 95% of adolescents reported experiencing at least one developmental stressor, and only 5% reported no stress for the listed stressors. The mean total developmental stress count was 11.70 (SD = 6.69) (out of a total of 30), and the mean stress level score (level 1–5) was 2.45 (SD = 0.73). In comparing the stress domains, physical appearance stress showed a significant sex difference, with females reporting higher stress levels (M = 2.73, SD = 1.29) than males (M = 2.40, SD = 1.21; *p* = 0.031).

### 3.2. Prediction for Adolescent Depression

#### 3.2.1. Using Adverse and Stress Experiences to Predict Adolescent Depression

We used a multidomain of ACEs and adolescent stress to predict the likelihood of developing adolescent depression. We tested the variation in prediction models by including different combinations of risk domains (see Table 2). Among the six tested ML approaches (described above), the random forest approach outperformed all other ML approaches (with the best AUC across models). Therefore, this paper focuses on the findings based on the random forest approach. For comparison, we presented random forest and logistic regression results in Table 2. Results for all the six tested ML models are presented in Appendix B. Overall, we found that when a single domain of adverse/stress experiences was included, ML models performed similarly to the logistic regression model (similar AUC and accuracy). However, when more than one domain of adverse/stress experiences were considered, ML models, in general, performed better than the logistic regression model (e.g., on AUC and accuracy, and specificity). When all 4 domains of adversity/stress experiences were considered (home, poverty, community/school ACEs, and adolescent developmental stress), ML/random forest models achieved the highest overall AUC (0.8361), accuracy (0.7825 or 78.3%), sensitivity (true positive rate 0.5175 or 51.8%), and specificity (true negative rate 0.8453 or 84.5%), exceeding the corresponding performance of logistic regression (AUC 0.8026, accuracy 74.4%, sensitivity 69.9%, and specificity 77.0%). Findings indicate that the predictability/accuracy in using adversity/stress experience to predict depression can achieve 78% accuracy, 52% true positive, or 85% true negative identification rate.

#### 3.2.2. Identifying Top-Ranking Risks in Predicting Adolescent Depression

Building upon the predictive modeling results in Section 3.2.1, we conducted a SHAP-based interpretability analysis to identify top-ranking risks. We used the best-performing random forest model that included all available domains of risks. We carried out the analysis in two ways—the global importance approach identifies the factors that are most influential overall in predicting depression risk, and the individual-level explanation approach identifies the single most influential predictor (top-1 factor) for each participant and the most frequently dominated individual-level risk profiles.

Using the global importance approach, we found that the top-15 and top-20 ranking models perform similarly (with all four performance indicators having prediction findings >70%), with only slightly better accuracy and sensitivity for the top-20 ranking model. The top-20 predictors included 6 measures from the ACEs (out of 19 items) and 14 measures from adolescent developmental stress (out of 30 items). The 6 ACEs include childhood neglect, abuse, neighborhood adversity, community violence, romantic relationship violence, and educational adversity/not being in school (see Table 3 for the items). The 14 adolescent developmental stress experiences include stressors related to academic stress, no leisure, physical appearance, physical health, peer pressure/social relationship stress, future uncertainty, money, and responsibility stress (Table 3 items labeled with AS/ASQ).

Using the individual-level explanation approach, we found slightly different rankings, but all of these were included in the top 20 based on the global importance approach. We found nearly half of the sample (48.2%) reported childhood neglect as the most influential risk factor, followed by schoolwork stress (8.5%), body change stress (6.9%), neighborhood adversity (5.7%), and IPV stress (4.6%).

## 4. Discussion

This paper applies ML and multiple domains of factors to predict adolescents’ risk of depression, as well as to identify the top-ranking risks that best predict adolescent risk of depression. This study offers proof of concept on how to develop ML models that have potential to identify adolescents at risk for depression early and prioritize public health resources to target key determinants to minimize the population’s risk for depression.

Our study contributes to new knowledge and methodology to inform new prevention, personalized care, and AI strategy development. Specifically, our study illustrates a new approach to prevention that moves toward a digital phenotyping effort and uses multidimensional and multifaceted real-world data sources to predict risk for depression and to early identify high-risk cases [38]. Our study is an attempt to understand whether ML can be applied to model dynamics of early and current adolescent stress experiences and accurately predict risks of depression for adolescents living in highly adverse LMIC contexts. Our findings illustrate ML can help us identify the top-ranking risks to predict adolescent depression relatively accurately (with 75% accuracy). If the ML models can be further validated and improved to achieve >80% or >90% accuracy, it would be possible to apply ML in school mental health screening and support the early prediction/identification of high-risk cases in low-resource school settings [39].

Our study also illustrates ML can be applied to help identify key determinants to screen and target. For example, in high-risk populations, we often focus on ACEs screening and ignore screening for adolescent developmental stress and community/school ACEs. Through a series of risk-domain prediction model comparisons, we found that focusing on a single domain of ACEs (home, community, or adolescent development) would not provide good accuracy in predicting adolescent depression. Only when community/school ACEs and adolescent stress were considered did the prediction accuracy became more accurate. Findings suggest that ACEs screening is insufficient; we need to consider a multifaceted approach to screening to better predict or prevent adolescent depression.

To be more targeted in prevention decision, we also applied ML to support intervention decision. In this study, we were able to identify top-15/top-20 risks (ACEs and developmental stress indicators) that were key to adolescent depression and could be used to predict adolescent depression reasonably accurately. The top-ranking risk findings suggest that school prevention efforts for our study population should target ACEs-related negative experiences (such as childhood neglect, abuse, neighborhood adversity, community violence, romantic relationship violence, and educational adversity/not being in school) and common developmental stress (such as academic stress, lack of leisure, worrying about physical appearance, physical health, peer pressure/social relationship stress, future uncertainty, money, and responsibility stress). Findings also suggest that schools may allocate and prioritize intervention resources to address these key risks/determinants/stressors to reduce the impacts of these risks on adolescents and to prevent their risk of depression. Future research should validate whether the ML algorithms can be applied to different populations to help identify key determinants that are most relevant to each population.

Additionally, our study illustrates the application of ML for supporting personalized care. Specifically, we applied an ML approach that allows us to identify top-ranking risks for everyone using the individual-level explanation approach (in contrast to the global importance approach). Such an approach allows us to identify different sets of top-15 or top-20 risk for different individuals, which can be applied to future personalized digital tool design and to provide personalized care based on differential risk priority.

Our systematic approach to developing and comparing ML models contributed to new methods and knowledge in the youth mental health field. The advanced ML approaches such as random forests can leverage complex patterns in the data that traditional linear models may not capture, making them particularly valuable in contexts where predictors interact in non-linear or synergistic ways. In mental health prediction model development, many studies have been based on demographic and data from wearable devices [40,41], but limited studies have considered the youth’s personal experience of stress. Our study is the first to focus on both adolescent adverse and stress experiences (i.e., including childhood and adolescent adversity and adolescent period of developmental stress experiences) in depression prediction. Our ML model development strategy is grounded in Social Determinants of Health (SDoH) [39,42], adverse childhood experiences (ACEs) [10,21], and adolescent developmental theory [22]. Our systematic approach to studying the predictability of the impact of adversity–stress factors on depression not only contributes to new knowledge and new ways to identify high-risk cases and key determinants, but also to validating an integrated risk/prediction theory (that includes a combination of risks from multiple theories). Our ML modeling allows us to identify unique distinct stress determinants (in ACEs and in adolescent developmental stress) that contribute to adolescent depression in LMIC community contexts.

Although our research has the potential to advance preventive mental health care, this study has several limitations. One, our outcome (depression) was based on cross-sectional data and a small sample. Future research should validate the prediction model based on longitudinal data and a larger sample. Two, the prediction model developed in this study was based on a community (school) sample and, mainly, for predicting adolescents to develop moderate to severe depression (in contrast to no and to mild depression). We did not use a more severe level clinical cutoff of 15 (moderate-severe to severe depression) for prediction because of a small clinical sample (<10% of our sample). For diagnosis prediction, more research is needed to include clinical cases in the ML prediction models. Three, our findings only inform one way to predict depression risk. Other prediction models that use different sets of predictors may yield better predictability. Our plan for the future is to continue to systematically explore prediction models for informing preventive intervention design, which may not be the same as predictive model development for clinical treatment purposes. For example, we plan to include adolescent individual-level skills factors (e.g., social-emotional learning skills, stress coping skills) and support factors (e.g., parenting style/support, peer support, community/school support) to better develop strategies to target skill building and support structures to reduce risks for depression. This line of research can be integrated into future AI-powered personalized preventive digital mental health tool design.

## 5. Conclusions

Innovative ML and modern predictive modeling approaches play a critical role in transforming modern preventive mental health care. This study illustrates how multidomain data can be used to identify individuals at risk for developing depression, as well as identify top-ranking risk factors to provide tailored interventions to specific populations and/or individuals to prevent depression. Among different ML approaches, ML/random forests appears to be a valuable approach to mining the data and can more accurately identify high-risk cases and key risks for depression. The data-driven approach of early identifying high-risk cases and top-ranking risks allows the better utilization of resources to target high-need cases and key risks/determinants that are most relevant to the targeted population. Our methods and findings can be helpful for LMICs and low-resource settings to plan for using limited resources more effectively. Our methods need to be further tested and validated with larger longitudinal and representative diverse samples before they can be applied to practice decisions.

## Figures and Tables

**Table 1:** Demographic and adversity and stress experience by adolescent sex.

	Total (n = 269)	Male (n = 136)	Female (n = 133)	*p*
Demographic	M (SD) or %	M (SD) or %	M (SD) or %	
Age	12.17 (1.04)	12.15 (0.99)	12.19 (1.08)	0.791
Religion				0.598
Muslim	5.7%	6.7%	0.7%	
Christian	94.3%	93.3%	95.3%	
Grade				0.338
5th Grade	30.5%	34.6%	26.3%	
6th Grade	33.1%	30.9%	35.3%	
7th Grade	36.4%	34.6%	38.3%	
Has Access to Internet				0.007
Yes	64.2%	56.3%	72.3%	
No	35.8%	43.7%	27.7%	
Have an Email account				0.447
Yes	2.6%	3.7%	1.5%	
No	97.4%	96.3%	98.5%	
Childhood Adversity Experiences	M (SD) or %	M (SD) or %	M (SD) or %	
(Count on the Number of Stress)				
ACEs-Home Adversity (0–10)	2.55 (2.08)	2.58 (2.18)	2.51 (2.00)	0.785
ACEs-Financial Adversity (0–3)	1.05 (1.02)	0.99 (1.02)	1.11 (1.02)	0.368
ACEs-Community Adversity (0–4)	1.13 (1.03)	1.09 (1.03)	1.19 (1.03)	0.394
ACEs-School Adversity (0–2)	0.56 (0.65)	0.60 (0.66)	0.52 (0.65)	0.310
ADS-Count Developmental Stress # (0–30)	11.70 (6.69)	11.58 (0.69)	11.82 (6.47)	0.772
ADS-Adolescent Stress Level (1–5) (Mean)	2.45 (0.73)	2.43 (0.75)	2.47 (0.70)	0.643
Mental Health-Depression				
PHQ9-A Total (Sum)(0–27)	7.71 (4.79)	9.40 (3.64)	10.44 (3.45)	0.339
PHQ9-A groups				0.420
Normal/minimal to Mild (0–9)	66.2%	69.1%	63.2%	
Moderate (10–14)	23.8%	22.1%	25.6%	
Moderately Severe (15–19)	9.3%	8.8%	9.8%	
Severe (20–27)	0.7%	0%	1.5%	

**Table 2:** Predictive model performance.

Models	Domains	AUC	Accuracy	Sensitivity	Specificity	Brier Score	ECE
Logistic Regression	(i) Home-ACEs	0.7543 (0.0678)	0.7009 (0.0580)	0.6537 (0.1164)	0.7259 (0.0806)	0.1915 (0.0319)	0.1382 (0.0365)
	(ii) Poverty-ACEs	0.6031 (0.0726)	0.5937 (0.0533)	0.6127 (0.1515)	0.5851 (0.0810)	0.2223 (0.0212)	0.1239 (0.0482)
	(iii) Community/School ACEs	0.7624 (0.0629)	0.7212 (0.0634)	0.6776 (0.1039)	0.7426 (0.0770)	0.1868 (0.0280)	0.1282 (0.0430)
	(iv) Ado. Developmental Stress	0.7088 (0.0717)	0.6584 (0.0623)	0.5830 (0.1185)	0.6968 (0.0856)	0.2131 (0.0302)	0.1770 (0.0398)
	(i) + (ii)	0.7434 (0.0610)	0.6893 (0.0551)	0.6560 (0.1248)	0.7071 (0.0696)	0.1957 (0.0306)	0.1489 (0.0373)
	(i) + (ii) + (iii)	0.7760 (0.0542)	0.6999 (0.0574)	0.6674 (0.1174)	0.7163 (0.0716)	0.1862 (0.0307)	0.1459 (0.0388)
	(i) + (ii) + (iii) + (iv)	0.8026 (0.0487)	0.7443 (0.0562)	0.6986 (0.1107)	0.7699 (0.0679)	0.1885 (0.0351)	0.1799 (0.0434)
Random Forests	(i)	0.7361 (0.0427)	0.6690 (0.0471)	0.5125 (0.1366)	0.7950 (0.0623)	0.1992 (0.0315)	0.1340 (0.0354)
	(ii)	0.6532 (0.0393)	0.5984 (0.0391)	0.2522 (0.1471)	0.8186 (0.1071)	0.2118 (0.0229)	0.1109 (0.0407)
	(iii)	0.7435 (0.0847)	0.6915 (0.0630)	0.5300 (0.1248)	0.8549 (0.0571)	0.1781 (0.0269)	0.1279 (0.0300)
	(iv)	0.7210 (0.0651)	0.6173 (0.0857)	0.3757 (0.1301)	0.8387 (0.0624)	0.1973 (0.0206)	0.1371 (0.0315)
	(i) + (ii)	0.7527 (0.0409)	0.6895 (0.0429)	0.5299 (0.1443)	0.8142 (0.0579)	0.1923 (0.0330)	0.1444 (0.0367)
	(i) + (ii) + (iii)	0.7734 (0.0403)	0.7306 (0.0433)	0.5423 (0.1706)	0.8212 (0.0551)	0.1856 (0.0305)	0.1405 (0.0403)
	(i) + (ii) + (iii) + (iv)	0.8361 (0.0438)	0.7825 (0.0421)	0.5175 (0.1263)	0.8453 (0.0589)	0.1750 (0.0261)	0.1337 (0.0418)

Note. Ado = adolescents. Model predictability was assessed using repeated five-fold cross-validation to ensure robust performance estimation and to minimize the influence of sampling variability. All reported metrics are presented as means with corresponding standard deviations, which reflect the stability of performance across folds. An AUC of 0.5 means random guessing, 1.0 is perfect, above 0.70 is commonly considered acceptable, with values above 0.80 regarded as good. An accuracy score above 70–75% is often interpreted as good for real-world clinical prediction models. For sensitivity and specificity, a level of 70% or greater is often targeted. Bolded rows are the models that had good prediction findings (>70%) across all four performance indicators. Six ML approaches were tested, but only the findings from the best ML models (random forests) are shown in this table.

**Table 3:** Top key risks for adolescent depression prediction and model performance.

**(a) Top 20. Risks Ranking (the Global Importance Approach).**					
	**Predictor Ranking**	**Item-Level Predictor Description**			
1	ACEs-Childhood Neglect	ACE3. Did you often feel that no one in your family loved you or thought you were important or special? Or your family didn’t look out for each other, feel close to each other, or support each other?			
2	ACEs-Neighborhood Adversity	ACE20. Do you agree that people in your neighborhood commonly disregard rules and laws, or do not care about what goes on in the neighborhood?			
3	AS-Academic Stress	ASQ2. Keeping up with schoolwork			
4	AS-Physical Appearance	ASQ7. Concern or worry about your body changes (changing too fast, too slow, or unexpected)			
5	AS-Physical Health	ASQ11. Having physical health concerns or challenges			
6	AS-Financial Pressure	ASQ26. Not enough money to buy the things you want			
7	ACEs-IPV	ACE17. In a romantic relationship, and being physically hurt, verbally or emotionally insulted, cursed, or threatened by your partner			
8	ACEs-Childhood Abuse (Emotion)	ACE1. Did a parent/guardian or other adult in the household often swear at you, insult you, put you down, or humiliate you, or act in a way that made you afraid you might be physically hurt			
9	ACEs-Community violence (Harassment)	ACE15. Were you ever verbally, physically, or sexually harassed (touched in an unwanted way)?			
10	AS-Romanic Relationship	ASQ21. Getting along with your boy/girlfriends in your romantic relationship			
11	AS-Role Responsibility	ASQ29. Having to take on added family responsibilities with growing older			
12	AS-Romantic Relationship	ASQ22. Making the relationship with your boy/girlfriend work			
13	ACEs-School Adversity	ACE18. Were you not in education or training in the past two years?			
14	AS-Peer Rejection Stress	ASQ18. Being ignored or rejected by peers, or not feeling part of the group			
15	AS-Future Uncertainty	ASQ24. Concern about your future			
16	AS-Social Relationship	ASQ17. Not getting along with your peers or friends			
17	AS-Leisure/Hobbies	ASQ4. Not getting enough time for leisure/fun or hobbies			
18	AS-Academic/School	ASQ3. Pressure for good grades			
19	AS-Peer/Social Stress	ASQ20. Experiencing bullying, cyberbullying, or discrimination			
20	AS-Peer Pressure	ASQ9. Not having things that other peers have			
**(b) Risk Ranking Model Performance**					
**Predictors**		**AUC**	**Accuracy**	**Sensitivity**	**Specificity**
Top 5		0.7452 (0.0534)	0.6955 (0.0483)	0.7447 (0.1103)	0.6720 (0.0630)
Top 10		0.8135 (0.0485)	0.6848 (0.0593)	0.8860 (0.0658)	0.5846 (0.0772)
Top 15		0.8359 (0.0491)	0.7476 (0.0537)	0.8136 (0.0932)	0.7158 (0.0745)
Top 20		0.8376 (0.0493)	0.7509 (0.0602)	0.8245 (0.0747)	0.7141 (0.0733)
All (items from 4 domains)		0.8361 (0.0438)	0.7825 (0.0421)	0.8043 (0.1158)	0.7718 (0.0329)

(a) Note. % reported in the table is the % reported moderately to very stressful. IPV = intimate partner/relationship violence. (b) Note. ML models using random forests are reported. Accuracy, sensitivity, and specificity scores above 70% are often interpreted as good for real-world clinical prediction models.

## Data Availability

The data presented in this study are available on request from the corresponding author due to privacy reasons.

## Appendix A. Study Measures Adaption

**Adaptation of Adverse Childhood and Adolescent Experiences (ACEs) Survey**

The Adverse Childhood Experiences International Questionnaire (ACE-IQ) [10,21] is a validated measure that can be used to assess an individual’s experiences of childhood adversities at home, such as child maltreatment and other family dysfunction and stressors. The measure has been validated with Kenya sample [21] and has been used globally [43].

*Rational for Adaptation*: To consider adverse experiences commonly experienced by adolescents in low-income-countries, we considered adapting the measure and including additional adverse items that are relevant to adolescents.

*Methods:* To do this, we carried out a qualitative study. Specifically, we first worked with community youth leaders (including adolescents and young adults, n = 30) in Kenya. We asked each youth leader to share one adolescent story (the story can be from their neighborhood, family, friend, or a fiction story that represents adolescents in their community) that described mental health challenges the adolescent experienced. In the story, we asked youth leaders to include the mental health or wellbeing problems/challenges that the adolescent encountered, how the adolescents saw/perceive the problems, how they felt (or emotion state), what others said about him/her (others’ perception about the adolescents), and what the adolescent did in this situation. We then carried out thematic analysis for the personas to identify common community and school stressors, as well as financial stressors experienced by adolescents. Based on the qualitative findings, we added 3 items for Poverty-ACEs and 7 items for Community/School ACEs (see Figure 1 for the added items). The tool was then reviewed by local educational stakeholders, parents, adolescents, and mental health researchers. Feedback was integrated and the tool was then used for this pilot testing. The final version used in this pilot study includes 9 added items—3 poverty-ACEs items and 6 Community/School ACEs items.

**Adaptation of Adolescent Stress Questionnaire (ASQ)**

Adolescent Development Stress was assessed using an adapted version of the Adolescent Stress Questionnaire (ASQ) [22] is a 58-item measure that assesses 10 areas of adolescent stress. Each item (stressor) is rated on a 5-point Likert scale (1 = not at all stressful/or irrelevant to me, 3 = moderately stressful, 5 = very stressful).

*Rational for Adaptation:* Because the questionnaire was too long and added burden to adolescents, we modified the questionnaire to a shorter version.

*Methods:* To shorten the measure, the study team selected items relevant to the LMIC context. We selected 3 most representative items in each area of stress (total of 10 areas). Based on the qualitative study described above, we also added a physical health stress item, as many adolescents experience physical health problems due to living in. The revised ASQ-K (for Kenya) captures common areas of adolescents’ stress, including academic/schoolwork stress, lack of leisure, physical appearance, peer comparison/comparative, physical health, home relationship, social relationship, romantic relationship, future uncertainty, financial pressure, and emerging adult responsibility stress (see Figure 1b for item information). The tool was then reviewed by local educational stakeholders, parents, adolescents, and mental health researchers. Feedback (mainly descriptive and language appropriateness) was integrated, and the tool was then used for this pilot testing.

## Appendix B

**Table A1:** Performance of the Six ML Predictive Models.

Models	Domains	AUC	Accuracy	Sensitivity	Specificity	Brier Score	ECE
Naive Bayes Classifier	(i)	0.7655 (0.0501)	0.7355 (0.0560)	0.5742 (0.1134)	0.8229 (0.0628)	0.1948 (0.0371)	0.1683 (0.0416)
	(ii)	0.6198 (0.0671)	0.6067 (0.0743)	0.1960 (0.1128)	0.8180 (0.0816)	0.2255 (0.0302)	0.1433 (0.0549)
	(iii)	0.7722 (0.0587)	0.7174 (0.0571)	0.5151 (0.1020)	0.8206 (0.0589)	0.1869 (0.0333)	0.1496 (0.0391)
	(iv)	0.7169 (0.0542)	0.6670 (0.0487)	0.6081 (0.1087)	0.6958 (0.0639)	0.2667 (0.0379)	0.2645 (0.0431)
	(i)+ (ii)	0.7594 (0.0537)	0.7311 (0.0570)	0.6010 (0.1049)	0.8030 (0.0590)	0.2028 (0.0380)	0.1794 (0.0415)
	(i) + (ii) + (iii)	0.7920 (0.0541)	0.7451 (0.0510)	0.6489 (0.1036)	0.7967 (0.0520)	0.2021 (0.0389)	0.1955 (0.0379)
	(i) + (ii) + (iii) + (iv)	0.8110 (0.0455)	0.7377 (0.0512)	0.7298 (0.1028)	0.7414 (0.0655)	0.2200 (0.0436)	0.2299 (0.0428)
Support Vector Machine with RBF Kernel	(i)	0.7196 (0.0750)	0.6812 (0.0680)	0.3237 (0.1132)	0.8673 (0.0608)	0.2008 (0.0318)	0.1414 (0.0401)
	(ii)	0.5711 (0.1145)	0.6387 (0.0630)	0.0510 (0.0930)	0.9413 (0.1021)	0.2284 (0.0213)	0.0815 (0.0490)
	(iii)	0.6914 (0.0863)	0.7345 (0.0557)	0.4272 (0.1285)	0.8955 (0.0495)	0.1927 (0.0251)	0.1034 (0.0381)
	(iv)	0.7395 (0.0523)	0.6923 (0.0470)	0.3208 (0.1078)	0.8857 (0.0547)	0.1934 (0.0171)	0.1283 (0.0344)
	(i) + (ii)	0.7358 (0.0652)	0.7057 (0.0652)	0.3841 (0.1322)	0.8702 (0.0610)	0.1954 (0.0325)	0.1295 (0.0417)
	(i) + (ii) + (iii)	0.7827 (0.0625)	0.7273 (0.0645)	0.4598 (0.1147)	0.8670 (0.0501)	0.1793 (0.0295)	0.1288 (0.0314)
	(i) + (ii) + (iii) + (iv)	0.8185 (0.0428)	0.7711 (0.0442)	0.6108 (0.1020)	0.8535 (0.0469)	0.1581 (0.0224)	0.1301 (0.0322)
Explainable Boosting Machines	(i)	0.7400 (0.0654)	0.7102 (0.0709)	0.3955 (0.1268)	0.8749 (0.0643)	0.1956 (0.0336)	0.1363 (0.0409)
	(ii)	0.6525 (0.0623)	0.6433 (0.0688)	0.0784 (0.0770)	0.9327 (0.0888)	0.2103 (0.0232)	0.1018 (0.0436)
	(iii)	0.7754 (0.0638)	0.7226 (0.0476)	0.4241 (0.1280)	0.8771 (0.0551)	0.1813 (0.0261)	0.1193 (0.0302)
	(iv)	0.7077 (0.0672)	0.6953 (0.0463)	0.4106 (0.1128)	0.8393 (0.0561)	0.2100 (0.0284)	0.1650 (0.0432)
	(i) + (ii)	0.7340 (0.0637)	0.7079 (0.0653)	0.4320 (0.1337)	0.8544 (0.0528)	0.1970 (0.0380)	0.1396 (0.0424)
	(i) + (ii) + (iii)	0.7860 (0.0711)	0.7353 (0.0728)	0.5193 (0.1443)	0.8492 (0.0498)	0.1810 (0.0423)	0.1506 (0.0432)
	(i) + (ii) + (iii) + (iv)	0.8136 (0.0392)	0.7592 (0.0414)	0.5724 (0.1178)	0.8546 (0.0407)	0.1799 (0.0308)	0.1673 (0.0403)
Histogram-based Gradient Boosting Machine	(i)	0.7206 (0.0531)	0.6723 (0.0642)	0.4344 (0.1135)	0.7970 (0.0447)	0.2069 (0.0361)	0.1588 (0.0506)
	(ii)	0.6692 (0.0632)	0.6276 (0.0600)	0.2438 (0.1612)	0.8322 (0.1112)	0.2119 (0.0247)	0.1193 (0.0366)
	(iii)	0.7692 (0.0632)	0.7174 (0.0573)	0.4795 (0.1288)	0.8411 (0.0576)	0.1830 (0.0289)	0.1423 (0.0362)
	(iv)	0.7133 (0.0557)	0.6989 (0.0435)	0.4077 (0.1302)	0.8474 (0.0484)	0.2158 (0.0255)	0.1928 (0.0380)
	(i) + (ii)	0.7209 (0.0632)	0.6997 (0.0691)	0.4758 (0.1391)	0.8137 (0.0696)	0.2063 (0.0391)	0.1790 (0.0475)
	(i) + (ii) + (iii)	0.7678 (0.0641)	0.7115 (0.0629)	0.5301 (0.1438)	0.8078 (0.0604)	0.1909 (0.0392)	0.1569 (0.0412)
	(i) + (ii) + (iii) + (iv)	0.7951 (0.0457)	0.7413 (0.0541)	0.5452 (0.1452)	0.8449 (0.0558)	0.1869 (0.0342)	0.1654 (0.0471)
XGBoost	(i)	0.6504 (0.0688)	0.6626 (0.0583)	0.4316 (0.0930)	0.7823 (0.0486)	0.2604 (0.0461)	0.2378 (0.0510)
	(ii)	0.6638 (0.0570)	0.6142 (0.0585)	0.2434 (0.1654)	0.8099 (0.1113)	0.2139 (0.0250)	0.1267 (0.0354)
	(iii)	0.7405 (0.0559)	0.7264 (0.0566)	0.5045 (0.1087)	0.8441 (0.0539)	0.1970 (0.0327)	0.1568 (0.0330)
	(iv)	0.7040 (0.0551)	0.6751 (0.0415)	0.4214 (0.1127)	0.8025 (0.0529)	0.2354 (0.0352)	0.2239 (0.0444)
	(i)+ (ii)	0.6739 (0.0677)	0.6782 (0.0669)	0.4596 (0.1142)	0.7914 (0.0570)	0.2459 (0.0499)	0.2277 (0.0569)
	(i) + (ii) + (iii)	0.7299 (0.0796)	0.7071 (0.0831)	0.5298 (0.1699)	0.8021 (0.0542)	0.2213 (0.0547)	0.2087 (0.0525)
	(i) + (ii) + (iii) + (iv)	0.7925 (0.0481)	0.7294 (0.0529)	0.5377 (0.1226)	0.8301 (0.0578)	0.2018 (0.0381)	0.1912 (0.0427)
Random Forests	(i)	0.7361 (0.0427)	0.6690 (0.0471)	0.5125 (0.1366)	0.7950 (0.0623)	0.1992 (0.0315)	0.1340 (0.0354)
	(ii)	0.6532 (0.0393)	0.5984 (0.0391)	0.2522 (0.1471)	0.8186 (0.1071)	0.2118 (0.0229)	0.1109 (0.0407)
	(iii)	0.7435 (0.0847)	0.6915 (0.0630)	0.5300 (0.1248)	0.8549 (0.0571)	0.1781 (0.0269)	0.1279 (0.0300)
	(iv)	0.7210 (0.0651)	0.6173 (0.0857)	0.3757 (0.1301)	0.8387 (0.0624)	0.1973 (0.0206)	0.1371 (0.0315)
	(i) + (ii)	0.7527 (0.0409)	0.6895 (0.0429)	0.5299 (0.1443)	0.8142 (0.0579)	0.1923 (0.0330)	0.1444 (0.0367)
	(i) + (ii) + (iii)	0.7734 (0.0403)	0.7306 (0.0433)	0.5423 (0.1706)	0.8212 (0.0551)	0.1856 (0.0305)	0.1405 (0.0403)
	(i) + (ii) + (iii) + (iv)	0.8361 (0.0438)	0.7825 (0.0421)	0.5175 (0.1263)	0.8453 (0.0589)	0.1750 (0.0261)	0.1337 (0.0418)

Note. Four domains of factors are: (i) Home-ACEs; (ii) Poverty-ACEs; (iii) Community/School ACEs; (iv) Ado. Developmental Stress.
